# Quality Evaluation of Ethambutol Hydrochloride Tablet Batches Available in Governmental Health Facilities of Jimma Town, Southwest Ethiopia

**DOI:** 10.1155/2021/9969157

**Published:** 2021-08-06

**Authors:** Meseret Yirdaw, Belachew Umeta, Yimer Mokennen

**Affiliations:** ^1^Department of Pharmacy, Jimma University Medical Center, Jimma, Oromia, Ethiopia; ^2^School of Pharmacy, Institute of Health, Jimma University, Jimma, Oromia, Ethiopia; ^3^Jimma University Laboratory of Drug Quality (JULaDQ), Jimma, Oromia, Ethiopia

## Abstract

**Background:**

The availability of poor-quality drugs on the drug market might favor the ineffectiveness of the drug and/antimicrobial resistance.

**Aim:**

To evaluate the quality of similar batches of ethambutol hydrochloride tablets available in different governmental health facilities of Jimma town, southwest Ethiopia.

**Methods:**

The World Health Organization checklist was used to inspect the storage area of health facilities and check medicines for the sign of counterfeit. The test was conducted as per the United States Pharmacopeia on six similar batches of ethambutol hydrochloride sampled from different governmental health facilities. Data were analyzed using SPSS version 20, and one-way ANOVA was used for comparing the dissolution profile and weight variation of batches.

**Results:**

Three health facilities did not comply with the storage area specifications for pharmaceuticals. No batches have shown any sign of counterfeit. All of the tablet batches tested complied with USP specifications for weight variation, percentage purity, and dissolution test. *Conclusions and Recommendation*. The entire tablet batches complied with the World Health Organization specification for packaging and labelling of pharmaceuticals. All tablet batches complied with the test for weight variation, purity of drug substance, and dissolution. Since some health facilities did not comply with at least one specification for storage of pharmaceuticals, regulatory agencies and stack holders are advised to inspect the health facilities to ensure appropriate storage of pharmaceuticals in health facilities.

## 1. Introduction

The quality of pharmaceutical products are affected by many factors; from those factors are stability of drug substance/s, the potential interaction between drug substance/s and excipients, manufacturing process, dosage form, packaging system, environmental conditions encountered during transport, storage and use, and length of time between manufacture and usage. Physicochemical and biological characteristics of drugs may be changed during storage leading to deterioration (physical, chemical, or microbial) and a decrease in their therapeutic use. In some cases, the deterioration may result in toxicity [1–4]. So, knowing the storage condition for individual drug product and maintaining the optimal storage condition was important throughout the shelf life of the drug to have the required therapeutic activity. Exposing pharmaceuticals to different environmental conditions such as temperature, humidity, sunlight, pH, moisture, and oxygen affect the rate and extent of drug degradation. Improper storage of pharmaceuticals also might lead to change in tablets hardness, friability, disintegration, and/dissolution rate which might in turn leads to change in bioavailability.

**Figure 1:**
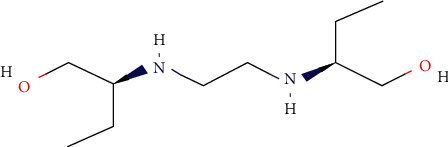
Chemical structure of ethambutol.

Chemically, ethambutol hydrochloride is a white crystalline powder. The drug is soluble in chloroform and sparingly soluble in water. Ethambutol hydrochloride exists as a dextrorotatory isomer of 2,2′-(ethylenediamine)-di-1-butanol dihydrochloride. The molecular weight of the drug is 204.31 g/mol [5, 6] (Figure 1).

According to the biopharmaceutical classification system, ethambutol hydrochloride was classified as a class III drug [7]. Class III drugs are rapidly dissolving, and permeability is the rate-controlling step in drug absorption. Rapid dissolution is desirable to maximize the contact time between the dissolved drug and absorption mucosa. Therefore, the duration of dissolution should be at least as stringent for class III [8]. Class III drugs exhibit high variability in rate and extent of absorption, but if dissolution is fast such that 85% of the drug dissolves in 15 minutes, the variation could be attributed to gastrointestinal transit, luminal contents, and membrane permeation rather than dosage form factors [9].

Substandard medicines pose a serious public health risk, especially in the developing world. These medicines are the product of poor manufacturing practices or improper storage or distribution practices that result in deterioration in the quality of the medicines. Substandard medicines may range from products that contain correct ingredients in incorrect proportions to products without active ingredients or with harmful substitutes. At their very best, these medicines are ineffective; at worst, they cause harm, creating drug-resistant pathogens or resulting in death [10]. Substandard medicines also pose a political risk, as they erode public confidence in health delivery systems.

## 2. Materials and Methods

### 2.1. Study Setting and Period

The study was conducted on similar batches of ethambutol hydrochloride sampled from different governmental health facilities present in Jimma town. Jimma town is 357 km southwest of Addis Ababa, the capital of Ethiopia, and the town has a specialized hospital, a general hospital, and five health centers. The samples were collected from all of the seven governmental health facilities. During sample collection, one facility did not have an ethambutol hydrochloride tablet. All the batches included in the study were within their shelf life at the time of the test (Table 1). The laboratory work was conducted in Jimma University Laboratory of Drug Quality (JULaDQ) from April to May 2019.

### 2.2. Instruments

HPLC (Agilent 1260 Series, Internal code: DQ.001), analytical balance (Mettler Toledo, internal code: DQ.047), RC-6D Dissolution Apparatus (Tian Jin Optical Instruments, China, internal code: DQ.017), UV-Vis spectrophotometer (Cecil Instruments, United Kingdom, internal code: DQ.019), water purification system (Thermo Scientific, USA, Model-7143, test internal code: DQ.051), and pH meter (Hungary, internal code: DQ.040), and 4.6 × 150 mm, 5 *μ*m column were used.

### 2.3. Chemical and Reagents

Triethylamine, phosphoric acid, methanol, ammonium hydroxide, HPLC-grade water, sodium hydroxide, acetonitrile, monobasic sodium phosphate, anhydrous sodium phosphate, and bromocresol green. The working standard of ethambutol was donated from the Ethiopian Food and drug Administration authority (EFDA).

### 2.4. Sampling Technique and Sample Collection

No specific sampling strategy was used to select the sample collection site as all of the governmental health facilities present in the town were included in the study. A simple random sampling technique was used to sample available ethambutol hydrochloride tablet batches from all government health facilities.

### 2.5. Quality Assessment Parameters

All of the tests were done in triplicate (*n* = 3), and the average value was used to report the data.

#### 2.5.1. Storage Area Characteristics of Tested Facilities

The storage areas for all of the governmental health facilities included in the study were evaluated according to the WHO checklist for inspection of storage facilities for pharmaceuticals [11].

#### 2.5.2. Physical Characteristics, Packaging and Labelling

The physical characteristics of the tablet batches were determined by physical inspection of shape, color, and the presence or absence of odor. Packaging and labelling information were checked using the modified World Health Organization (WHO) checklist designed to carry out visual inspection of medicines for signs of counterfeiting [12].

#### 2.5.3. Weight Variation Test

Randomly selected twenty tablets from each batch were weighed individually with a calibrated analytical balance. The average weight for each tablet batch was determined, and the percentage deviation from the average weight was calculated. Finally, the percentage deviation was compared with the USP acceptance criteria (no more than two (2) individual masses deviate by >5% of the average tablet weight, and none deviate by more than 10%) [13]. The percentage deviation from the average was calculated using the following formula:

(1) $$\begin{array}{c} \%\text{ deviation}=\frac{\text{tablet weight }-\text{ average weight}}{\text{average weight}}\times 100. \end{array}$$

#### 2.5.4. Dissolution Test

*(1) Phosphate Buffer Preparation*. Phosphate buffer was prepared by dissolving 38 g of monobasic sodium phosphate and 20 gm of anhydrous dibasic sodium phosphate in 1000 mL water.

*(2) Bromocresol Green Solution Preparation*. Bromocresol green solution was prepared by dissolving 200 mg of bromocresol green in 30 mL water and 6.5 mL of 0.1 N sodium hydroxide. The solution was then diluted with phosphate buffer to 500 mL, mixed, and the PH was adjusted to 4.6 ± 0.1 by 0.1 N hydrochloric acids.

*(3) Standard Solution Preparation*. The concentration of 0.1 mg/mL of ethambutol hydrochloride reference standard (RS) was prepared by dissolving 10 mg of USP ethambutol hydrochloride in 100 mL of water.

*(4) Dissolution Test Condition*. The dissolution test was conducted according to the USP monograph on six tablets of each batch using USP Apparatus I operated at 100 rpm. The dissolution medium was 900 mL phosphate buffer (pH = 6.8) maintained at 37°C ± 0.5°C. USP 2015 specifies at a single time of 45 minutes, not less than 75% labelled amount needs to be dissolved13. However, 5 mL of sample was withdrawn at 5, 15, 30, 45, and 60 minutes to study the dissolution profile of the tablets. And a fresh 5 mL dissolution medium was used to replace the withdrawn sample after each sampling.

*(5) Test Procedure*. Into three separate glass-stoppered 50 mL centrifuge tubes pipet, 1 mL of water was added as a blank, 1 mL standard solution as a reference, and 1 mL of sample solution as a test. To each tube, 5 mL of bromocresol green solution was added and mixed, and to each tube, 10 mL of chloroform was added; then, the stopper was inserted and shaked vigorously. The mixture was allowed to separate, and the aqueous upper layer was discarded. The three chloroform layer was filtered using cotton pledgets. Then, the corresponding absorbance readings of diluted filtrates were taken by a UV-visible spectrophotometer at a wavelength of 415 nm. Finally, the amount of ethambutol hydrochloride dissolved was determined from the calibration curve.

**Figure 2:**
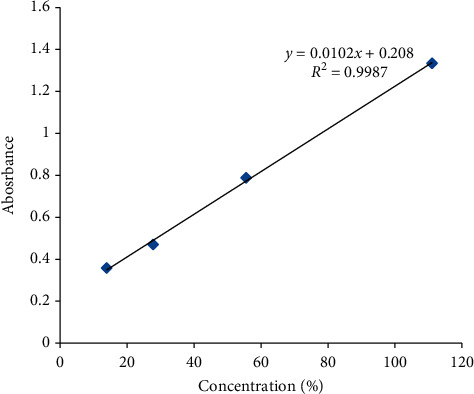
Calibration curve for dissolution study of ethambutol hydrochloride 100 mg tablet batches.

*(6) Construction of Calibration Curve for Dissolution Study*. The calibration curve was constructed in a similar manner with the preparation of samples for dissolution study as described above. The curve was constructed by preparing four concentration levels of 100%, 50%, 25%, and 12.5% of the stock solution. The calibration had an equation of *Y* = 0.0102*X* + 0.208, where “*Y*” is the absorbance and “*X*” is the concentration in *μ*g/mL. The curve showed that there was a linear relationship between the concentration of the tested samples and the absorbance values with *r*^2^ = 0.9987 (Figure 2). By using the equation obtained from the calibration curve, the percentage release values of samples taken at times 5, 15, 30, 45, and 60 minutes were calculated.

#### 2.5.5. Amount of Active Ingredient

*(1) Buffer Solution Preparation*. The buffer solution was prepared by mixing 1 mL of trimethylamine with 1 L of water, and the pH was adjusted to 7.0 using phosphoric acid. The solution was then filtered and degassed.

*(2) Mobile Phase Preparation*. Mobile phase (buffer/ACN, 50/50% v/v) was prepared and filtered with Millipore filter paper (0.45 *μ*m).

*(3) Standard Solution Preparation*. The concentration of 0.3 mg/mL standard solution was prepared by dissolving 30 mg ethambutol hydrochloride working standard in 100 mL of water.

*(4) Preparation of Samples*. Twenty tablets from each batch of ethambutol hydrochloride were taken randomly, weighed individually and finely powdered. A portion of powder equivalent to about 30 mg of ethambutol hydrochloride was taken and transferred into 100 mL volumetric flask. 20 mL of water was added and sonicated for 15 minutes to ensure complete dissolution of the powder. This solution was diluted with water to volume. Finally, the solution was filtered with a filter paper having a porosity of 0.45 *μ*m, and the first 10 mL portion was discarded. The percentage quantity of ethambutol hydrochloride in the portion of tablets was calculated using the following formula:

(2) $$\begin{array}{c} \%\text{ purity}=100C\;\left(\frac{r_{u}}{r_{s}}\right), \end{array}$$

where *r*_u_ is the peak response from the sample solution; *r*_s_ is the peak response from the standard solution; and *C* is the concentration of ethambutol hydrochloride from the reference standard RS (mg/mL).

*(5) Chromatographic Condition*. HPLC system equipped with analytical column (4.6 × 150 mm 5 *μ*m) and UV-Vis Diode Array Detector (DAD) with a detection wavelength of 200 nm, the flow rate of 1.0 mL/min, and injection volume of 50 *μ*L were used. The mobile phase composition was acetonitrile and buffer solution (50 : 50% v/v) with a pH of 7.00.

### 2.6. Data Analysis

Microsoft Excel 2010 and SPSS version 20 software programs were used for statistical analysis of analytical data. Statistically significant differences were considered when *p* < 0.05, and one-way ANOVA was used for comparison of weight variation and dissolution profile of tested tablets with each other. Mean dissolution times were calculated for each of the tablet batches tested by using KinetDS software. DE is the area under the dissolution curve within a time range [14].

## 3. Results

All of the tablet batches were imported from foreign countries.

### 3.1. Storage of the Drug in Health Facilities Included in the Study

The storage area of each of the governmental health facilities included in the study was inspected by using the WHO checklist for inspection of storage area for pharmaceuticals, and three of the facilities (F4, F5, and F6) failed to comply with WHO guidelines for at least two parameters for storage of pharmaceuticals (Table 2).

### 3.2. Physical Inspection, Packaging, and Labelling Characteristics

The physical inspection characteristics of the studied tablet batches showed that all of them had a uniform white color, undamaged, and did not have any odor. The packaging and labelling of all tablet batches meet the minimum requirement required by the World Health Organization for the packaging and labelling of pharmaceuticals.

### 3.3. Weight Variation and Friability Test

F1-ET001 and F6-ET006 had the highest and lowest mean weight, respectively. All of the tablet batches complied with USP acceptance criteria for weight variation (Table 3).

Statistical analysis conducted using one-way ANOVA at 95% Confidence Interval (CI) revealed that there were significant differences (*p* < 0.001) in mean weight among samples mean weight of all batches tested (Table 3). In addition to one-way ANOVA, the Tukey multiple comparisons test was performed at 95% CI to pin out the source of difference between each of the tablet batches tested. The test revealed that except some batches all of the batches had similar mean tablet weights with each other (Table 4).

### 3.4. Assay Test

**Figure 3:**
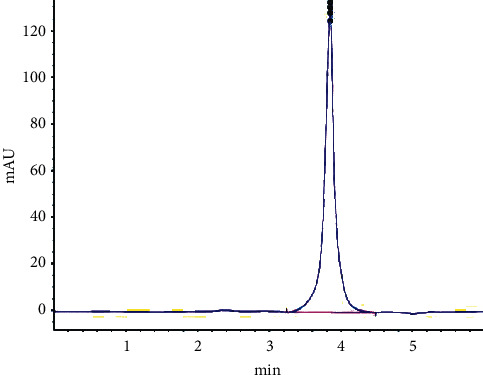
Representative chromatogram for analysis of ethambutol hydrochloride 100 mg tablet batches.

Representative chromatogram is shown in Figure 3. All of the tested tablet batches were within the acceptable limit. The assay result of ethambutol hydrochloride tablet batches tested ranges from 102.7% to 104.01%. F4-ET004 had highest assay value, and F2-ET002 had the lowest assay value with 104.0% and 102.7% purity, respectively (Table 3).

### 3.5. Dissolution Profile

**Figure 4:**
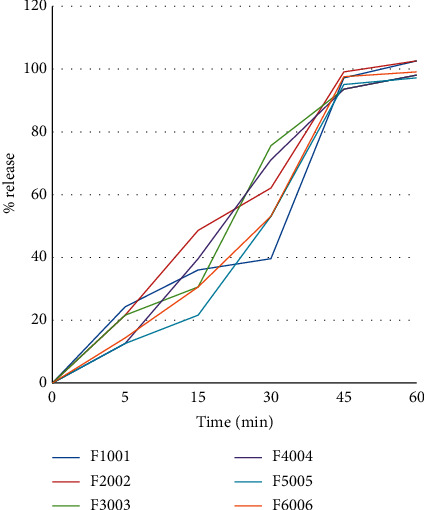
Dissolution profile of ethambutol hydrochloride 100 mg tablet batches samples included in the study.

All of the tested tablet batches released the necessary amount of active pharmaceutical ingredient (API) at a pharmacopeial specified time of 45 minutes (Figure 4). One-way ANOVA conducted at 95% CI revealed no statistically significant difference in the mean dissolution between each batch tested. To augment the one-way ANOVA, Tukey's multiple comparisons were conducted and revealed that the entire tested tablet batches had similar dissolution profiles with each other (Table 5).

### 3.6. Mean Dissolution Time

F5-ET005 had the highest mean dissolution time, and F2-ET002 had the lowest mean dissolution time (Table 3).

## 4. Discussion

The results of visual inspection of packaging and labelling information of the tested samples did not show any signs of spurious, falsely labelled, falsified, or counterfeit products as defined by the World Health Organization. This suggests that the packaging and labelling information of each ethambutol HCl tablet batches sampled from health facilities in Jimma town were in line with WHO guidelines on the packaging and labelling of pharmaceutical products.

The results of the weight variation test showed that the mean weight ranges from 118.94 to 120.00 mg. The acceptance criteria for the weight variation test as per USP stated that not more than two individual masses deviate by greater than 5% of the average tablet weight, and none deviate by more than 10%. As per these criteria, all of the batches complied with the specification. Weight variation test is used to test the degree of uniformity in drug substances among dosage units [15]. Moreover, statistical analysis conducted using one-way ANOVA at 95% CI revealed that there were significant differences (*p* < 0.001) among sample mean weight of all batches tested. To ensure the consistency of dosage units, each unit in a batch should have a drug substance contained within a narrow range around the label claim.

The assay result of ethambutol hydrochloride tablet batches ranged from 102.7% to 104.01%. Of the tested tablet batches, all complied with the United States Pharmacopeia specifications (%LC: 95–105). The study conducted in Nigeria reported that from three tablets tested, none of them complied with the test for percentage purity as stated on British Pharmacopeia [16]. The other study done in Senegal in 2015 reported that all of the tablets tested complied with acceptance criteria for purity of the tablet test [17]. The study done in Syria to investigate the effect of moisture and temperature on furosemide tablets (40 mg) revealed that high humidity and temperature (RH = 75% and 40°C) decreased in hardness and content of furosemide tablets (less than 90%) and increased in friability (more than 1%) in all studied brands [18].

The rate of release for ethambutol hydrochloride in this study ranged from 95.1% to 99.1% within the first 45 minutes. The rate of drug released should not be less than 75% of the labelled amount for ethambutol hydrochloride at 45 minutes as per the United States Pharmacopoeia. The result of one-way ANOVA at 95% CI showed that there were no statistically significant differences in the release pattern among sample mean weight of different tablets sampled from different health facilities (*p*=0.620).

Mean dissolution time (MDT) is used to characterize the drug release rate from the dosage form and the retarding efficiency of the polymer. A higher value of MDT indicates the lowest rate of drug substance release from the dosage form, which leads to slow onset of action and higher drug retaining ability of the polymer and vice versa. The MDT value is also a function of polymer loading, polymer nature, and physicochemical properties of the drug molecule [19]. Accordingly, F5-ET005 had the highest retaining ability of the polymer. Therefore, F5-005 might have a slow onset of action for this batch.

## 5. Conclusions and Recommendation

Only half of the health facilities meet all of the requirements for the storage area of pharmaceuticals, and the entire tablet batches were in line with the modified World Health Organization checklist for packaging and labelling of pharmaceuticals for the sign of counterfeit. All tablet batches complied with specifications for weight variation, assay, and dissolution test. To ensure appropriate storage of pharmaceuticals, the regulatory agencies and stack holders were strongly advised to inspect and regulate the storage of drugs within the health facilities.

### 5.1. Limitation of the Study

The study was done on single tablet brand with similar batch numbers. So, it does not represent the general survey to assess the quality of different brands of ethambutol hydrochloride tablets. However, the finding will give an insight for future researchers and regulatory bodies to evaluate the effect of pharmaceutical storage on the quality of drugs.

## Figures and Tables

**Table 1:** Some information of ethambutol hydrochloride 100 mg tablet batches tested.

Sample code	Manufacturer	Country of origin	Man. date	Exp. date	Coated/uncoated
F1-ET001	ET001M1	CO	09/2015	08/2019	Film coated
F2-ET002	ET002M2	CO	09/2015	08/2019	Film coated
F3-ET003	ET003M3	CO	09/2015	08/2019	Film coated
F4-ET004	ET004M4	CO	09/2015	08/2019	Film coated
F5-ET005	ET005M5	CO	09/2015	08/2019	Film coated
F6-ET006	ET006M6	CO	09/2015	08/2019	Film coated

F : code for facilities; ET : ethambutol hydrochloride; and CO : country of origin.

**Table 2:** Checklist for evaluation of storage area of governmental health facilities from which the tablet batches were sampled.

Inspection checklist for storage area of pharmaceuticals						
Facility code	F1	F2	F3	F4	F5	F6
Are there locks which are working in the storage area	√	√	√	√	√	√
Storage and shelves area are clean (no dust or litter)	√	√	√	√		
No evidence of pests seen in the area	√	√	√	√	√	√
There is a ceiling	√	√	√	√	√	√
There are windows that can be opened or there are air vents	√	√	√	√	√	√
No direct sunlight should enter the area, glass window pane painted white, or with curtains/blinds to protect against sunrays	√	√	√	x	x	x
Area free from moisture (leaking drains and taps). Drugs should not be stored directly on the floor	√	√	√	√	x	x
There is a separate storage and dispensing area for issuing drugs	√	√	√	√	√	√
Drugs are sorted in systematic way (alphabetical, first expired, first out)	√	√	√	√	√	√
There is stock record system	√	√	√	√	√	√
There is a cold storage with temperature chart	√	√	√	x	x	x

√: comply; x: did not comply; F: code for health facility; and F4, F5, and F6: did not monitor the temperature of storage area.

**Table 3:** Physicochemical characteristics (weight variation and assay test) for ethambutol hydrochloride 100 mg tablet batches included in the study (*n* = 3).

Code for batches	Weight variation (mean ± SD)	UB	LB	*p* value	Assay (%) (mean ± SD)	MDT
F1-ET001	120.00 ± 1.36901	119.36	120.65	*p* < 0.001	104.0 ± 1.89	25.68
F2-ET002	119.71 ± 2.02959	118.77	120.66		102.7 ± 1.62	20.62
F3-ET003	119.76 ± 1.50971	119.06	120.47		103.81 ± 1.36	21.08
F4-ET004	118.94 ± 1.83058	118.09	119.80		104.01 ± 1.39	21.31
F5-ET005	119.04 ± 1.88721	118.16	119.92		103.00 ± 1.93	31.94
F6-ET006	114.11 ± 2.12634	113.12	115.11		104.00 ± 1.84	27.45

SD = standard deviation; UB = upper bound; LB = lower bound; MDT = mean dissolution time.

**Table 4:** Tukey's multiple comparisons test for weight variation of ethambutol hydrochloride tablet batches tested with each other.

Code for batches	Comparators	Mean difference (code-comparator)	Std. error	Sig.	95% confidence interval	
					Lower bound	Lower bound
F1-ET001	F2-ET002	0.29	0.57	0.996	−1.37	1.95
	F3-ET003	0.24	0.57	0.998	−1.42	1.90
	F4-ET004	1.06	0.57	0.439	−0.60	2.72
	F5-ET005	0.96	0.57	0.551	−0.70	2.62
	F6-ET006	5.89^*∗*^	0.57	<0.001	4.23	7.55

F2-ET002	F3-ET003	−0.05	0.57	1.000	−1.71	1.61
	F4-ET004	0.77	0.573	0.760	−0.89	2.43
	F5-ET005	0.67	0.57	0.851	−0.99	2.33
	F6-ET006	5.6^*∗*^	0.57	<0.001	3.94	7.26

F3-ET003	F4-ET004	0.82	0.57	0.708	−0.84	2.48
	F5-ET005	0.72	0.57	0.808	−0.94	2.38
	F6-ET006	5.65^*∗*^	0.57	<0.001	3.99	7.31
	F5-ET005	−0.100	0.57	1.000	−1.76	1.56

F4-ET004	F6-ET006	4.83^*∗*^	0.57	<0.001	3.17	6.49
F5-ET005	F6-ET006	4.93^*∗*^	0.57	<0.001	3.27	6.59

^*∗*^The mean difference is significant at the 0.05 level. F: code for health facilities; ET: ethambutol hydrochloride.

**Table 5:** Tukey's multiple comparisons test for weight variation of ethambutol hydrochloride tablet batches tested with each other.

Code for batches	Comparators	Mean difference (code-comparator)	Std. error	Sig.	95% confidence interval	
					Lower bound	Lower bound
F1-ET001	F2-ET002	−6.86	20.58	0.999	−70.49	56.77
	F3-ET003	−3.96	20.58	1.000	−67.59	59.67
	F4-ET004	−3.06	20.58	1.000	−66.69	60.57
	F5-ET005	22.14	20.58	0.886	−41.49	85.77
	F6-ET006	17.38	20.58	0.956	−46.26	81.01

F2-ET002	F3-ET003	2.90	20.58	1.000	−60.73	66.53
	F4-ET004	3.80	20.58	1.000	−59.83	67.43
	F5-ET005	29.00	20.58	0.721	−34.63	92.63
	F6-ET006	24.24	20.58	0.843	−39.39	87.87

F3-ET003	F4-ET004	0.90	20.58	1.000	−62.73	64.53
	F5-ET005	26.10	20.58	0.799	−37.53	89.73
	F6-ET006	21.34	20.58	0.901	−42.29	84.97

F4-ET004	F5-ET005	25.20	20.58	0.821	−38.43	88.83
	F6-ET006	20.44	20.58	0.916	−43.19	84.07

F5-ET005	F6-ET006	−4.76	20.58	1.000	−68.39	58.87

F: code for facilities; ET: ethambutol hydrochloride.

## Data Availability

The data used to support the findings of this study are available from the corresponding author upon request.
